# A Descriptive Analysis of Overviews of Reviews Published between 2000 and 2011

**DOI:** 10.1371/journal.pone.0049667

**Published:** 2012-11-15

**Authors:** Lisa Hartling, Annabritt Chisholm, Denise Thomson, Donna M. Dryden

**Affiliations:** 1 Alberta Research Centre for Health Evidence, Department of Pediatrics, University of Alberta, Edmonton, Alberta, Canada; 2 Cochrane Child Health Field, Department of Pediatrics, University of Alberta, Edmonton, Alberta, Canada; University of Illinois-Chicago, United States of America

## Abstract

**Background:**

Overviews of systematic reviews compile data from multiple systematic reviews (SRs) and are a new method of evidence synthesis.

**Objectives:**

To describe the methodological approaches in overviews of interventions.

**Design:**

Descriptive study.

**Methods:**

We searched 4 databases from 2000 to July 2011; we handsearched *Evidence-based Child Health: A Cochrane Review Journal*. We defined an overview as a study that: stated a clear objective; examined an intervention; used explicit methods to identify SRs; collected and synthesized outcome data from the SRs; and intended to include only SRs. We did not restrict inclusion by population characteristics (e.g., adult or children only). Two researchers independently screened studies and applied eligibility criteria. One researcher extracted data with verification by a second. We conducted a descriptive analysis.

**Results:**

From 2,245 citations, 75 overviews were included. The number of overviews increased from 1 in 2000 to 14 in 2010. The interventions were pharmacological (n = 20, 26.7%), non-pharmacological (n = 26, 34.7%), or both (n = 29, 38.7%). Inclusion criteria were clearly stated in 65 overviews. Thirty-three (44%) overviews searched at least 2 databases. The majority reported the years and databases searched (n = 46, 61%), and provided key words (n = 58, 77%). Thirty-nine (52%) overviews included Cochrane SRs only. Two reviewers independently screened and completed full text review in 29 overviews (39%). Methods of data extraction were reported in 45 (60%). Information on quality of individual studies was extracted from the original SRs in 27 (36%) overviews. Quality assessment of the SRs was performed in 28 (37%) overviews; at least 9 different tools were used. Quality of the body of evidence was assessed in 13 (17%) overviews. Most overviews provided a narrative or descriptive analysis of the included SRs. One overview conducted indirect analyses and the other conducted mixed treatment comparisons. Publication bias was discussed in 18 (24%) overviews.

**Conclusions:**

This study shows considerable variation in the methods used for overviews. There is a need for methodological rigor and consistency in overviews, as well as empirical evidence to support the methods employed.

## Introduction

Overviews of systematic reviews (overviews) are a relatively new method of evidence synthesis. [1] While systematic reviews (SRs) are successful at bringing together multiple studies in a rigorous fashion, a limitation is that often comparative data across different interventions are lacking, and these data are critical for informed decision-making by clinicians, policy-makers, and others. [2] To address this shortcoming, overviews compile data from multiple SRs relevant to a single health problem in a format and with methods analogous to SRs.

Overviews provide a single synthesis of all relevant evidence and may be useful for therapeutic and policy decision-making regarding the disease or condition in question. [1] For example, an overview of interventions for bronchiolitis can serve as a comprehensive “friendly front end” to the evidence, meaning that the reader does not have to assimilate the data from seven separate SRs on different therapeutic options. [3]–[10] Further, they may be produced more quickly because they are based on existing SRs, thereby providing more timely evidence for decision-making. [2] Overviews could be a useful resource for policy-makers in developing clinical practice guidelines, decision support systems, and drug formularies.

As overviews represent a relatively new research design, their methodology has not undergone extensive study. Current guidance is largely driven by personal experience and “good practice” rather than empirical evidence. [2] Research and guidelines are required to advance the methods of this emerging methodology to ensure that they are comprehensive, free of bias, and represent the most valid results for end-users. The purpose of this paper is to provide a first step to inform the methodology for overviews. The objectives were to 1) identify all overviews of health care interventions published in the health care literature between 2000 and 2011; and 2) describe the overviews with respect to the number published and their methodological approaches.

## Methods

We searched four databases from 2000 to July 2011: MEDLINE (OVID), EMBASE (OVID), DARE (OVID), and SCOPUS. The search strategy for MEDLINE was as follows:

1. (overview adj3 reviews).tw or (overview adj2 review*).
2. (umbrella adj5 review*).tw.
3. (systematic adj1 overview*).tw.
4. (systematic adj3 overview*).tw.
5. (overview adj2 cochrane adj2 reviews).tw.
6. (systematic adj1 reviews).ti.
7. ((appraisal or analysis or results) adj2 systematic adj review*).tw.
8. (meta-synthesis or (meta adj synthesis)).tw.
9. (meta-review or (meta adj review)).tw.
10. or/1–9
11. limit 10 to (english language and humans and yr = “2000–2011”)

The searches for other databases are available from the authors. We hand-searched *Evidence-based Child Health: A Cochrane Review Journal* (EBCH), as we were aware that this journal publishes overviews in almost every issue.

We defined an overview as a review designed to compile evidence from multiple SRs addressing the effects of two or more interventions for a single condition or health problem and to summarize their results for important outcomes as determined by authors of the overview. We included overviews if they stated a clear objective; examined an intervention; used explicit methods to identify relevant SRs; collected and synthesized outcome data from the SRs or the studies they contained; and intended to include only SRs or meta-analyses. Inclusion criteria were as follows:

1. Did the study state a clearly formulated question or objective?
2. Did the question or objective involve an intervention?
3. Did the study use explicit methods to identify relevant systematic reviews (i.e., systematic searching)?
4. Did the study collect and analyse outcome data from the relevant systematic reviews or the studies they contained?
5. Did the study intend to include only systematic reviews or meta-analyses?

Two researchers independently screened and categorized studies as ‘include’, ‘exclude’, or ‘unclear’. The full text of studies categorized as ‘include’ or ‘unclear’ by either researcher was retrieved. Two researchers independently applied the eligibility criteria to the full text articles. A third researcher was consulted when study eligibility was unclear.

One researcher extracted data with verification by a second researcher. We extracted the following information: year of publication; funding source; condition(s); intervention(s); objective (whether clearly stated); inclusion criteria (whether clearly stated); search methods (number of databases, dates of search provided, search strategy or key words provided, additional search methods reported, any restrictions [Cochrane only, language, year, published literature]); study selection methods (whether reported; if reported, did 2 authors independently screen and complete full text review with consensus procedure for agreements, or other approach); numbers included (SRs, primary studies, participants); details on inclusion/exclusion (whether included SRs of randomized controlled trials [RCTs] only, list of included SRs provided, list of excluded SRs provided); data extraction methods (whether reported; if reported, did 2 authors independently data extraction, or other approach); methods for quality/risk of bias assessment of primary studies (was quality/risk of bias extracted from original SRs, tools used and approach [e.g., 2 independent assessors]; was quality/risk of bias assessed by overview authors, tools used and approach); methods for quality/risk of bias assessment for SRs (whether preformed, tool used, approach used [e.g., 2 independent assessors]); grading of evidence (was quality of evidence extracted from original SRs, tools used and approach [e.g., 2 independent assessors]; was grade or strength of evidence assessed by overview authors, tools used and approach); data synthesis (were characteristics of included SRs provided, including data on participants, interventions, and outcomes; was a quantitative analysis performed; were outcomes specified in methods and determined a priori); and whether publication bias discussed.

We conducted a descriptive analysis using frequencies and percentages to summarize the variables collected. We provide overall results, and we provide results separately for the EBCH overviews and non-EBCH overviews.

## Results

**Figure 1:**
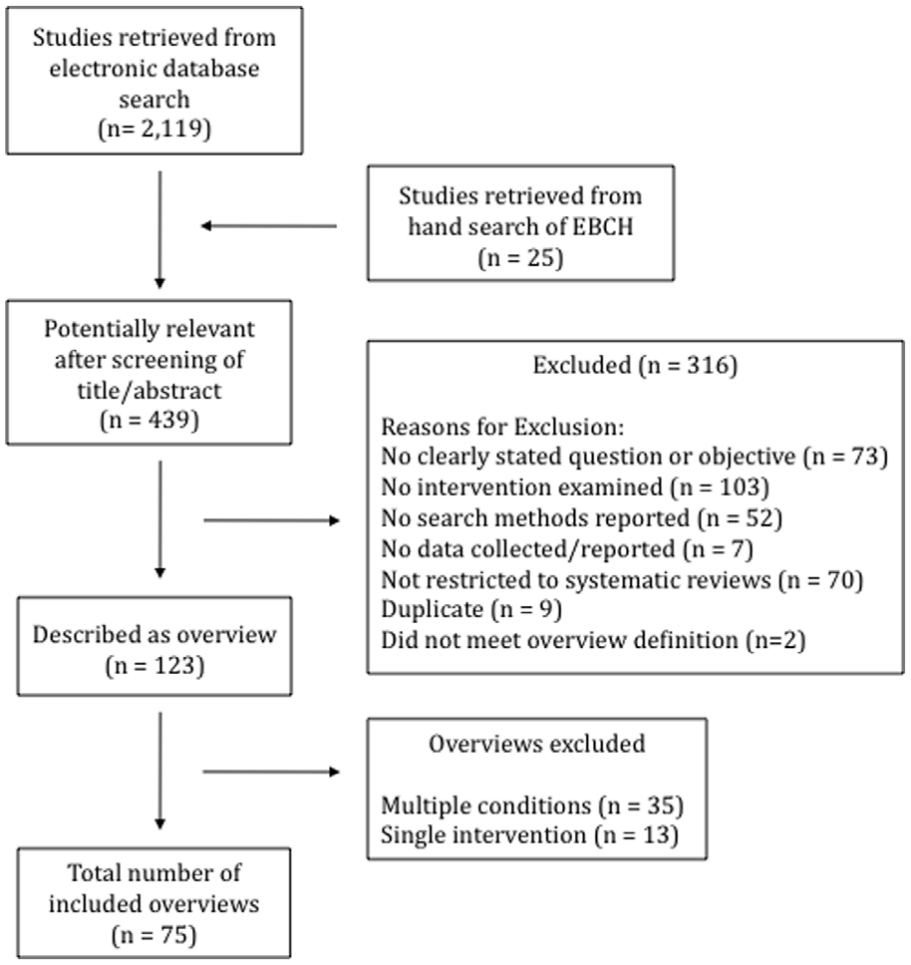
Flow of studies through the screening and selection process.

**Figure 2:**
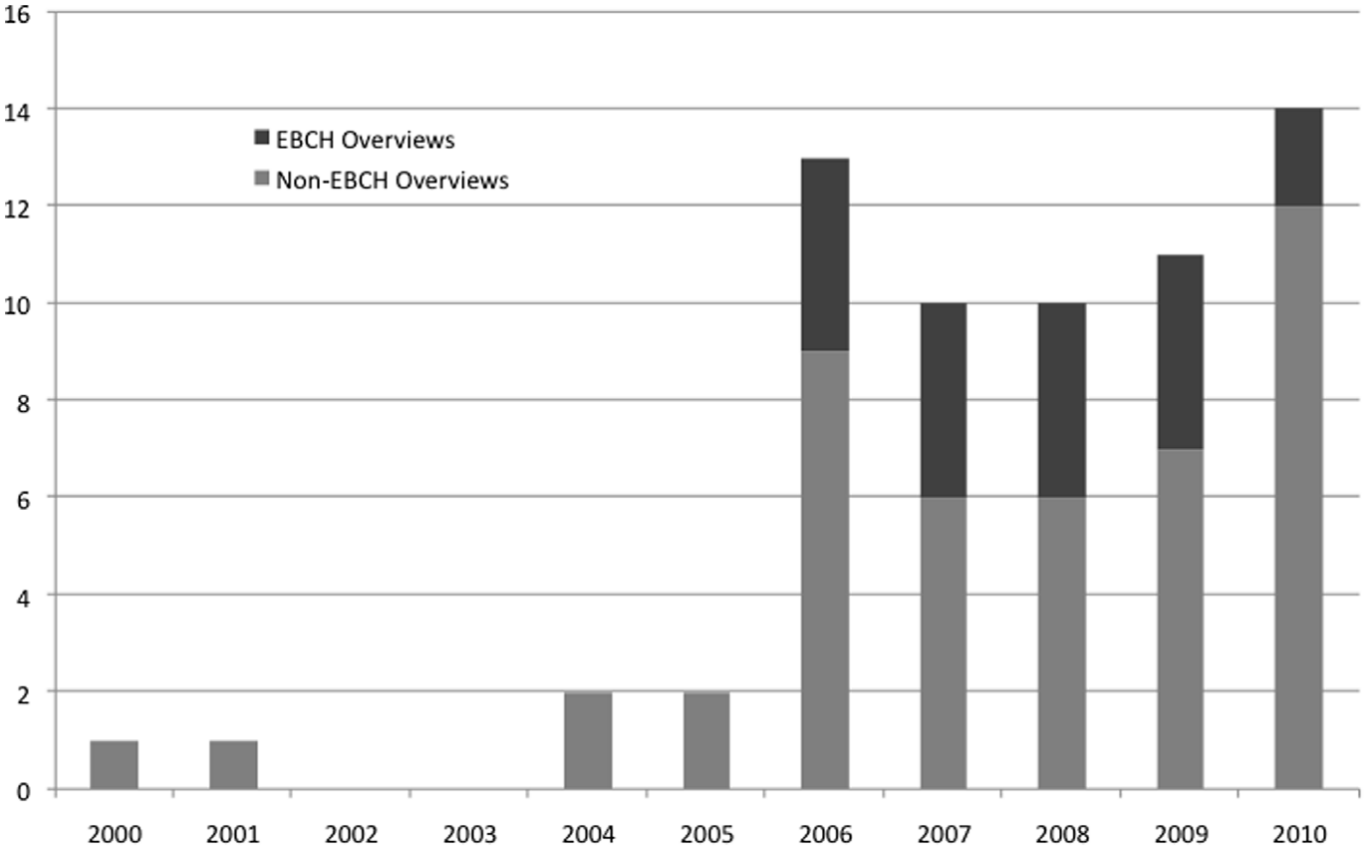
Number of overviews published by year, 2000–2010*. *2011 was not included in the figure as the full year was not captured (search completed July 2011).

The search yielded 2,245 citations. Of these, 414 were considered potentially relevant and 75 overviews met the inclusion criteria (Figure 1); a list of included overviews is available from the authors. Of the 75 overviews, 24 (32%) were published in EBCH. The number of overviews increased over time from 1 in 2000 to 14 in 2010 (Figure 2). The increase in 2006 could be due in part to the regular publication of overviews in EBCH starting in that year. The interventions examined in the overviews were most often combinations of pharmacological and non-pharmacological (n = 29, 38.7%), with others examining only pharmacological (n = 20, 26.7%) or only non-pharmacological (n = 26, 34.7%). The overviews included a median of 6 SRs (range 0 to 153) and 56 primary studies (range 0 to 2,062). The number of study participants was recorded in 39 overviews and ranged from 411 to more than 300,000. Thirty-two overviews included only RCTs (43%). The majority provided a list of included SRs (n = 68, 91%); however, few provided a list of excluded SRs (n = 17, 23%).

A variety of terms was used to describe the study design: analysis of systematic reviews, guidelines based on systematic review evidence, meta-review, overview, overview of Cochrane and non-Cochrane reviews, overview of Cochrane reviews, overview of Cochrane systematic reviews, overview of review, overview of reviews, overview of systematic reviews, review, review of systematic reviews, summary of Cochrane, summary of systematic reviews, synopsis of Cochrane systematic reviews, systematic meta-review, systematic review, systematic review of reviews, systematic review of systematic reviews, systematic umbrella review, umbrella review. The most frequent labels were overview of reviews (n = 20, 27%), overview of systematic reviews (n = 10, 13%), umbrella review (n = 8, 11%), review of systematic reviews (n = 6, 8%), overview of Cochrane systematic reviews (n = 4, 5%), review (n = 3, 4%), and analysis of systematic reviews (n = 3, 4%).

Thirty-three overviews searched at least 2 databases (44%). The majority of overviews reported the years and databases searched (n = 46, 61%), and provided key words (n = 58, 77%). In addition to electronic databases, the most frequent sources to identify studies were reference lists of related or included studies (n = 18, 24%), and consultation with the relevant Cochrane group (n = 9, 12%). Inclusion criteria were clearly stated in the majority of overviews (n = 65, 87%). Thirty-nine overviews included Cochrane SRs only (52%). In 11 overviews, the search was not restricted by language of publication (15%), in 16, language restrictions were applied (21%), and in 48 restrictions for language were not mentioned (64%). Thirty-seven overviews clearly stated that searches were not limited by date restrictions (49%). Eleven overviews reported a search for unpublished or grey literature (15%).

Methods for selecting studies to include were reported in 37 overviews (49%). Two reviewers independently screened and completed full text review in 29 overviews (39%). Other methods used were one author undertaking study selection (n = 3, 4%), one author screened titles and abstracts and two authors screened full text (n = 1, 1%), one author assessed and a second confirmed (n = 1, 1%), one author screened and if in doubt a second author was asked to check (n = 1, 1%). In two overviews (3%), the description of selection methods did not mention the number of reviewers involved.

Methods of data extraction were reported in 45 overviews (60%). Two authors independently extracted data in 35 overviews (47%). Other methods included data extraction by one author (n = 4, 5%), data extraction by one author who discussed results with a second author (n = 2, 3%), data extraction was completed by one author and verified by a second (n = 2, 3%), data extraction by one author and some data reviewed by a biostatistician and second author (n = 1, 1%). In one case, the authors mentioned use of a data extraction form but did not report the number of authors who extracted the data.

Methodological quality of individual studies was extracted from the original SRs in 27 overviews (36%). In one overview (1%) authors assessed quality or risk of bias for the individual studies. Twenty-eight (37%) overviews assessed the quality of included SRs using 9 different tools. Four overviews (5%) used more than 1 tool. The Oxman and Guyatt Overview Quality Assessment Questionnaire (OQAQ) [11] was used most often (n = 9, 27%); other tools used were AMSTAR (n = 7, 21%), QUORUM guidelines (n = 3, 9%), Jadad decision algorithm for interpretation of discordant reviews (n = 1, 3%), Methodological Quality Checklist (n = 1, 3%), the Index of the Scientific Quality of Research Overviews (n = 1, 3%) and the Systematic Review Appraisal Sheet of the Centre for Evidence Based Medicine, University of Oxford (n = 1, 3%). In one case, the authors reported the level of research design (i.e., hierarchy of evidence) (3%). Additionally, 9 overviews (27%) assessed quality of SRs using various individual components. The methods for quality assessment of the SRs were reported in 23 of the 28 overviews (82%) that conducted quality assessment. In most cases, double independent assessment was performed (n = 21, 28%). The quality of the body of evidence was assessed in 13 overviews (17%). In most cases the GRADE tool was used (n = 12, 16%). The quality of the body of evidence was extracted from the SRs in one overview (1%).

Most overviews provided a narrative or descriptive analysis of the included SRs. Fifty-nine overviews (79%) described the characteristics of the included SRs, including data on participants, interventions, and outcomes. The outcomes of interest were mentioned in the methods section of 27 overviews (36%); the outcomes were specified as having been determined a priori in 16 overviews (21%). A quantitative analysis was conducted in only 2 cases (3%): one overview conducted indirect analyses and another conducted mixed treatment comparisons. Publication bias was discussed in 18 overviews (24%).

Thirty overviews (40%) reported a total of 35 different sources of funding: government (n = 17, 49%), industry (n = 6, 17%), foundations (n = 4, 11%), partnership between government, charity and industry (n = 1, 3%) and internal funding (n = 2, 6%). In five overviews, the authors reported that no funding had been received (14%). The remaining overviews (n = 45, 60%) did not report on source of funding.

Table 1 compares the 24 overviews published in EBCH with overviews published in the general health care literature. Overviews published in EBCH were generally smaller with a median of 5 SRs (range 2 to 11) and 35 primary studies (range 4 to 230). With one exception, EBCH overviews restricted their searches to the Cochrane Database of Systematic Reviews and included only Cochrane SRs. In almost all EBCH overviews, the objectives and inclusion criteria were clearly stated (96%). Half of the EBCH overviews reported including SRs of RCTs only. All provided a list of SRs but only 38% provided a list of excluded SRs. In 79% of overviews, the methods for data extraction were reported with two reviewers involved in half (50%). In the majority of EBCH overviews (75%), quality or risk of bias assessments of individual trials were extracted from the original SRs. Quality assessment of the SRs was performed in only 2 overviews (8%) and different tools were used in each. Assessing the quality of evidence using GRADE was performed in 4 EBCH overviews (17%). Characteristics of the participants, interventions, and outcomes for each SR were described in 22 overviews (92%). All EBCH overviews performed a qualitative or narrative synthesis, with none conducting quantitative analysis of data. Publication bias was discussed in 4 of these overviews (17%), and the source of funding was reported in 4 (17%).

**Table 1:** Description of overviews published in the health care literature and in *Evidence-based Child Health: A Cochrane Review Journal* (n and % reported unless otherwise indicated).

	Health Care Literature, N = 51	*Evidence-based Child Health,* N = 24	Total, N = 75
**Interventions Included**			
Pharmacological only	14 (27.5)	6 (25.0)	20 (26.7)
Non-pharmacological only	22 (43.1)	3 (12.5)	25 (33.3)
Both	15 (29.4)	15 (62.5)	30 (40.0)
**Objectives Clearly Stated**	51 (100)	23 (95.8)	74 (98.7)
**Inclusion Criteria Clearly Stated**	42 (82.4)	23 (95.8)	65 (86.7)
**Comprehensive Search Strategy**			
≥2 Databases	32 (62.7)	1 (4.2)	33 (44.0)
Years *and* databases reported	37 (72.5)	9 (37.5)	46 (61.3)
Search strategy/Key words	35 (68.6)	23 (95.8)	58 (77.3)
**Search Restrictions**			
Restricted to Cochrane only	16 (31.4)	23 (95.8)	39 (52.0)
Restricted by year	16 (31.4)	1 (4.2)	17 (22.7)
Restricted to published literature	11 (21.6)	21 (87.5)	32 (42.7)
**Selection Methods**			
Reported	27 (52.9)	10 (41.7)	37 (49.3)
2 authors	23 (45.1)	6 (25.0)	29 (38.7)
1 author	n/a	3 (12.5)	3 (4.0)
Other	4 (7.8)	1* (4.2)	5 (6.7)
**Number Included**			
Systematic reviews, median (range)	8 (0–153)	5 (2–11)	6 (0–153)
Primary studies, median (range)	78 (0–2,062)	35 (4–230)	56 (0–2,062)
Study participants, range	411 to>300,000	618 to 18,581	411 to>300,000
**Characteristics of Included SRs**			
SRs of RCTs only	20 (39.2)	12 (50.0)	32 (42.7)
List of SRs provided	44 (86.3)	24 (100)	68 (90.7)
List of excluded studies	11 (21.6)	9 (37.5)	20 (26.7)
**Data Extraction Methods**			
Reported	26 (51.0)	19 (79.2)	45 (60.0)
2 authors	21 (41.2)	12 (50.0)	33 (44.0)
1 author	1 (2.0)	4 (16.7)	5 (6.7)
Other methods	4 (7.8)	3 (12.5)	7 (9.3)
**Quality and Risk of Bias Assessments of Individual studies**			
Extracted from original SRs	9 (17.6)	18 (75.0)	27 (36.0)
-methods reported	6 (11.8)	2 (8.3)	8 (10.7)
-double independent assessment	–	2 (8.3)	2 (2.7)
Performed by overview authors	0 (0.0)	2 (8.3)	2 (2.7)
-methods reported	–	1 (4.2)	1 (1.3)
-double independent assessment	–	1 (4.2)	1 (1.3)
**Quality Assessment of Systematic Reviews**			
Assessed by overview authors	26 (51.0)	2 (8.3)	28 (37.3)
Reported use of specific tools	26 (51.0)	2 (8.3)	28 (37.3)
-Oxman and Guyatt	8 (15.7)	0 (0.0)	8 (10.7)
-AMSTAR	6 (11.8)	1 (4.2)	7 (9.3)
-QUOROM	3 (5.9)	0 (0.0)	3 (4.0)
Methods reported	21 (41.2)	2 (8.3)	23 (30.7)
-double independent assessment	19 (37.3)	2 (8.3)	21 (28.0)
**Grading of Evidence**			
Extracted from SRs	1 (2.0)	0 (0.0)	1 (1.3)
Performed by overview authors	9 (17.6)	4 (16.7)	13 (17.3)
Reported use of specific tool (all used GRADE)	8 (15.7)	4 (16.7)	12 (16.0)
Methods			
-not reported	2 (3.9)	2 (8.3)	4 (5.3)
-double independent assessment	5 (9.8)	1 (4.2)	6 (8.0)
-single independent assessment	2 (3.9)	1 (4.2)	3 (4.0)
**Synthesis**			
Included characteristics on participants, interventions and outcomes	37 (72.5)	22 (91.7)	59 (78.7)
**Analysis**			
Quantitative analysis across SRs	2 (3.9)	0 (0.0)	2 (2.7)
**Publication Bias**			
Discussed	14 (27.5)	4 (16.7)	18 (24.0)
**Source of Funding**			
Reported	26 (51.0)	4 (16.7)	30 (40.0)
-industry *(n and % of those reporting funding)*	4 (13.3)	2 (40.0)	6 (20.0)
-government *(n and % of those reporting funding)*	15 (50.0)	2 (40.0)	17 (22.7)
-institutional *(n and % of those reporting funding)*	3 (10.0)	1 (20.0)	4 (13.3)
-internal *(n and % of those reporting funding)*	2 (6.7)	0	2 (6.7)
-other *(n and % of those reporting funding)*	1 (3.3)	0	1 (3.3)
-no funding *(n and % of those reporting funding)*	5 (16.7)	0	5 (16.7)

* All authors in consultation with relevant Cochrane Review Group.

## Discussion

This study shows considerable variation in the methods used for overviews. Overviews have the potential to be a useful tool for the translation of health evidence and decision-making. This study demonstrates that overviews contain a substantial amount of information—the median number of SRs and primary studies included were 6 and 56, respectively, reflecting data from 400 to 300,000 participants. However, guidance and standards are required to ensure the methodological rigor and consistency of overviews. Moreover, empirical evidence is required to support the methods employed.

The methods of searching within our sample of overviews varied depending on whether the intent was to include both Cochrane and non-Cochrane SRs. In general, the methods for searching when including both Cochrane and non-Cochrane SRs were comprehensive and well reported, although there is room for improvement (e.g., searching more than 2 databases, reporting the years and databases searched, reporting a search strategy or key words). The methods for other aspects of the overviews such as study selection, data extraction, and quality assessment were less often reported and did not conform to standard SR methodology of involving two independent reviewers in order to minimize selection bias.

A starting point for overview methods would be to replicate methods used in SRs. The Institutes of Medicine in the US and The Cochrane Collaboration have recently released standards for the conduct of SRs [12], [13] and these can serve as a guide. Reporting guidelines have emerged in recent years for many study designs. For example, CONSORT and PRISMA are endorsed by medical journal editors worldwide to guide reporting of RCTs and SRs, respectively. [14], [15] Similar guidelines need to be established and followed for overviews. Priority should be placed on consistent terminology of this study design (i.e., overviews of reviews) to guide authors, peer reviewers, journal editors, and those involved with indexing efforts. In Appendix S1, we list the mandatory standards developed by The Cochrane Collaboration [13] and discuss their applicability to overviews, and we present the PRISMA reporting guidelines for SRs and meta-analyses [15] and their applicability to overviews. These resources can provide some guidance for authors of overviews; however, this guidance will need further evaluation and refinement with expert input as our collective experience in producing overviews increases.

As with other forms of evidence synthesis, the utility of overviews will be largely dependent on the availability and quality of SRs and ultimately the trials or other studies that they include. Deficiencies in the methodological quality at each level can compromise the results and conclusions of an overview and ultimately its utility for decision-makers. The assessment of methodological quality or risk of bias of primary studies is a key step in SRs and there has been an extensive investment in developing and evaluating tools to this end. There has been some work to develop similar tools to assess the quality of SRs. To date, the most commonly used tool in overviews is the OQAQ; [11] however, AMSTAR has been recommended to assess the methodological quality of SRs in other contexts. [16]–[18] We also found that reporting guidelines were used inappropriately to assess methodological quality. Methodological guidance is needed to ensure that the quality of both the SRs and the primary studies they include is adequately assessed and incorporated into the results and conclusions of overviews. A challenge with relying on quality assessment performed in the original SRs is that often different tools or components are used; therefore, it can be difficult to compare evidence across SRs. While there is evidence showing that the methodological quality, or risk of bias, of primary studies can affect the magnitude and direction of effect estimates, [19] the relationship between methodological quality of SRs and the effect estimates or conclusions of the SRs has not been established.

A methodological step that has been incorporated into SRs more recently has been to assess the quality of the body of evidence. The Cochrane Collaboration has endorsed the GRADE approach to assess quality of evidence and detailed guidance on the use of GRADE has been published. [20] Few overviews in our sample described the quality of evidence, likely because this process has been implemented more recently. However, a number of overview authors applied the GRADE tool. Guidance is needed to ensure appropriate use and interpretation of the GRADE tool/criteria when assessing quality of evidence based on SRs rather than primary studies (for which the tool was designed). As the uptake of GRADE increases within SRs, overview authors should report the quality of evidence as assessed by the SR authors. The SR authors are in the best position to assess the quality of evidence given their familiarity with the study-level data including the details that feed into the GRADE domains, such as risk of bias, consistency, precision, directness, and reporting bias. This is important information to present in an overview so that the reader can understand, and make decisions based on, the quality of evidence supporting the different interventions being compared.

The vast majority of overviews described the relevant SRs in a qualitative or narrative manner. Only two overviews conducted quantitative analysis of the data: one conducted indirect analyses and one conducted mixed treatment comparisons. Emerging methods for analysis such as mixed treatment comparisons (or network meta-analysis) allow for comparisons among all relevant interventions for a condition and may allow comparisons among interventions that have not been directly compared in head-to-head studies. Overviews provide a unique context to conduct advanced statistical analyses in order to make comparisons across treatments that might not be included in single SRs and to make use of indirect data. However, guidance is needed for employing advanced statistical methods in the context of overviews, in particular the feasibility and challenges of conducting such analyses based on data available in the SRs versus the need to gather data from the primary studies.

A third of our sample of overviews was published in EBCH. This on-line journal was launched in 2006 with a mandate to profile timely and topical child-relevant Cochrane evidence. As part of this, the founding editors felt that the synthesis function carried out by overviews of reviews would be a valuable service to readers. An overview has been included in almost every issue (4 issues/year from 2006 to 2010, 6 issues/year from 2011 to present). The methods for producing the overviews in EBCH have been informed by the Cochrane Comparing Multiple Interventions Methods Group (formerly named the Umbrella Reviews Working Group). Given that overviews represent a new approach to evidence synthesis, the methods advocated by this group have evolved over time and this variation confirms the need for guidance for all overviews including those published in EBCH.

### 

#### Strengths and Limitations

This research provides a detailed descriptive analysis of the methods used in overviews. One of the key limitations is the definition we used for overviews. We used the definition provided by the Cochrane Collaboration [1] and sought input from one of the co-conveners of the Cochrane methods group for overviews of reviews. However, our definition was restricted to multiple interventions for a single condition. During our screening process we identified reviews that examined a single intervention for multiple conditions, e.g., acupuncture for a variety of conditions. These were not included in our analysis. A further limitation is that we may not have identified all overviews because of the variable terminology that has been used in the literature. Therefore, our findings with regards to terminology used to describe “overviews” and methods used may not be comprehensive or wholly representative. Finally, we only searched for overviews reported in English. Despite these limitations, we feel that this work reflects the variation in methodology used in overviews and clearly underscores the need for guidance in terms of methods and reporting for this emerging publication type.

### Conclusions

There is an increasing number of overviews of reviews being published in the health care literature. There is substantial variation in the methodological approaches used in overviews, and deficiencies in reporting of key methodological steps. Guidance is needed for the conduct and reporting of overviews, as well as empirical evidence to support the methodological approaches used. A starting point would be to follow well-recognized recommendations for methods and reporting of SRs.

## Supporting Information

Appendix S1 **Table 1** **) Methodological standards for Cochrane Intervention Reviews and their application to overviews of reviews; Table 2) Preferred Reporting Items for Systematic Reviews and Meta-analyses (PRISMA) and their applicability to overviews of reviews.**
(DOCX)
