# Supplementary material for: A Descriptive Analysis of Overviews of Reviews Published between 2000 and 2011
Source: PLoS One. 2012 Nov 15;7(11):e49667. doi: 10.1371/journal.pone.0049667 (PMC3499476; doi:10.1371/journal.pone.0049667)
Supplement: Appendix S1 — Table 1 ) Methodological standards for Cochrane Intervention Reviews and their application to overviews of reviews; Table 2) Preferred Reporting Items for Systematic Reviews and Meta-analyses (PRISMA) and their applicability to overviews of reviews. (DOCX) [file pone.0049667.s001.docx]

**Appendix S1**

Table 1. Methodological standards for Cochrane Intervention Reviews and their application to overviews of reviews (http://www.editorial-unit.cochrane.org/sites/editorial-unit.cochrane.org/files/uploads/MECIR_conduct_standards%202.1.pdf)

| **Item No.* †** | **Item name** | **Standard** | **Applicability to overviews of reviews** |
| --- | --- | --- | --- |
| Setting the research question(s) to inform the scope of the review | | | |
| 1 | Formulating review questions | Ensure that the review question and particularly the outcomes of interest, address issues that are important to stakeholders such as consumers, health professionals and policy makers. | Directly applicable |
| 2 | Pre-defining objectives | Define in advance the objectives of the review, including participants, interventions, comparators and outcomes. | Directly applicable |
| 3 | Considering potential adverse effects | Consider any important potential adverse effects of the intervention(s) and ensure that they are addressed. | Applicable. Overview authors should identify important outcomes including adverse effects and comment if any are not addressed or reported in the included SRs. If not addressed or reported in the SRs, overview authors need to decide whether to examine the primary studies to see if relevant outcomes were reported at the primary study level but not extracted at the SR level. |
| Setting eligibility criteria for including studies in the review | | | |
| 5 | Pre-defining unambiguous criteria for participants | Define in advance the eligibility criteria for participants in the studies. | Directly applicable |
| 7 | Pre-defining unambiguous criteria for interventions and comparators | Define in advance the eligible interventions and the interventions against which these can be compared in the included studies. | Directly applicable |
| 8 | Clarifying role of outcomes | Clarify in advance whether outcomes listed under ‘Criteria for considering studies for this review’ are used as criteria for including studies (rather than as a list of the outcomes of interest within whichever studies are included) | Directly applicable |
| 9 | Pre-defining study designs | Define in advance the eligibility criteria for study designs in a clear and unambiguous way, with a focus on features of a study’s design rather than design labels. | Directly applicable; need to define what is considered a SR. |
| 12 | Excluding studies based on publication status | Include studies irrespective of their publication status, unless explicitly justified. | Directly applicable |
| 13 | Changing eligibility criteria | Justify any changes to eligibility criteria or outcomes studied. In particular, post hoc decisions about inclusion or exclusion of studies should keep faith with the objectives of the review rather than with arbitrary rules. | Directly applicable |
| 14 | Pre-defining outcomes | Define in advance which outcomes are primary outcomes and which are secondary outcomes. | Directly applicable |
| Planning the review methods at protocol stage | | | |
| 19 | Planning the search | Plan in advance the methods to be used for identifying studies. Design searches to capture as many studies as possible meeting the eligibility criteria, ensuring that relevant time periods and sources are covered and not restricting by language or publication status. | Directly applicable |
| 20 | Planning the assessment of risk of bias in included studies | Plan in advance the methods to be used for assessing risk of bias in included studies, including the tool(s) to be used, how the tool(s) will be implemented, and the criteria used to assign studies, for example, to judgements of low risk, high risk and unclear risk of bias. | Applicable. Overview authors should determine whether they will extract risk of bias assessments from the included SRs or conduct risk of bias assessments on the primary studies themselves. Overview authors should determine how they will handle discrepancies in approaches to risk of bias assessments across SRs. Overview authors should determine whether and how they will assess methodological quality of the included SRs. |
| 21 | Planning the synthesis of results | Plan in advance the methods to be used to synthesize the results of the included studies, including whether a quantitative synthesis is planned, how heterogeneity will be assessed, choice of effect measure (e.g. odds ratio, risk ratio, risk difference or other for dichotomous outcomes), and methods for meta-analysis (e.g. inverse variance or Mantel Haenszel, fixed-effect or random effects model). | Applicable. Overview authors should determine how they will present the data from included SRs and whether they will re-analyze data to provide consistency (e.g., choice of effect measure, method of analysis). |
| 22 | Planning subgroup analyses | Pre-define potential effect modifiers (e.g. for subgroup analyses) at the protocol stage; restrict these in number; and provide rationale for each. | Applicable. Overview authors should specify subgroups of interest and determine whether they will conduct additional analyses if subgroups of interest are not examined or reported in the included SRs. |
| Searching for studies | | | |
| 24 | Searching key databases | Search the Cochrane Review Group's Specialized Register (internally, e.g. via the Cochrane Register of Studies, or externally via CENTRAL). Ensure that CENTRAL and MEDLINE (e.g. via PubMed) have been searched (either for the review or for the Review Group’s Specialized Register). | Applicable. Overview authors should search The Cochrane Library (i.e., Cochrane Database of Systematic Reviews and Database of Abstracts of Reviews of Effectiveness) and may wish to consult relevant Cochrane Review Groups for a listing of reviews. |
| 32 | Structuring search strategies for bibliographic databases | Inform the structure of search strategies in bibliographic databases around the main concepts of the review, using appropriate elements from PICO and study design. In structuring the search, maximize sensitivity whilst striving for reasonable precision. Ensure correct use of the AND and OR operators. | Directly applicable |
| 33 | Developing search strategies for bibliographic databases | Identify appropriate controlled vocabulary (e.g. MeSH, Emtree, including 'exploded' terms) and free-text terms (considering, for example, spelling variants, synonyms, acronyms, truncation and proximity operators). | Directly applicable |
| 35 | Restricting database searches | Justify the use of any restrictions in the search strategy on publication date, publication format or language. | Directly applicable |
| 36 | Documenting the search process | Document the search process in enough detail to ensure that it can be reported correctly in the review. | Directly applicable |
| 37 | Rerunning searches | Rerun or update searches for all relevant databases within 12 months before publication of the review or review update, and screen the results for potentially eligible studies. | Directly applicable |
| Selecting studies into the review | | | |
| 39 | Making inclusion decisions | Use (at least) two people working independently to determine whether each study meets the eligibility criteria, and define in advance the process for resolving disagreements. | Directly applicable |
| 40 | Excluding studies without useable data | Include studies in the review irrespective of whether measured outcome data are reported in a ‘usable’ way. | Directly applicable |
| 41 | Documenting decisions about records identified | Document the selection process in sufficient detail to complete a PRISMA flow chart and a table of ‘Characteristics of excluded studies’. | Directly applicable |
| 42 | Collating multiple reports | Collate multiple reports of the same study, so that each study rather than each report is the unit of interest in the review. | Directly applicable (e.g., SR published in Cochrane Library and peer-reviewed journal; published and unpublished version of he same SR). |
| Collecting data from included studies | | | |
| 43 | Using data collection forms | Use a data collection form, which has been piloted. | Directly applicable |
| 44 | Describing studies | Collect characteristics of the included studies in sufficient detail to populate a table of ‘Characteristics of included studies’. | Directly applicable |
| 46 | Extracting outcome data in duplicate | Use (at least) two people working independently to extract outcome data from reports of each study, and define in advance the process for resolving disagreements. | Directly applicable |
| 47 | Making maximal use of data | Collect and utilize the most detailed numerical data that might facilitate similar analyses of included studies. | Applicable. Overview authors should extract detailed data from meta-analyses when available that will facilitate comparisons across SRs. |
| 50 | Choosing intervention groups in multiarm studies | If a study is included with more than two intervention arms, include in the review only intervention and control groups that meet the eligibility criteria. | Overview authors should be aware of how SR authors have handled such studies. |
| 51 | Checking accuracy of numeric data in the review | Compare magnitude and direction of effects reported by studies with how they are presented in the review, taking account of legitimate differences. | Applicable. Caution is needed when comparing interventions that have not been formally compared in either direct or indirect analyses. |
| Assessing risk of bias in included studies | | | |
| 52 | Assessing risk of bias | Assess the risk of bias for each included study. | Determine a priori whether overview authors will assess the methodological quality of included SRs and what tool will be used. |
| 53 | Assessing risk of bias in duplicate | Use (at least) two people working independently to apply the risk of bias tool to each included study, and define in advance the process for resolving disagreements. | Applicable based on assessing methodological quality of SRs. |
| 54 | Supporting  judgements of risk of bias | Justify judgements of risk of bias (high, low and unclear) and provide this information in the ‘Risk of bias’ tables (as ‘Support for judgement’). | Applicable based on assessing methodological quality of SRs. |
| 61 | Incorporating assessments of risk of bias | If randomized trials have been assessed using one or more tools in addition to the Cochrane ‘Risk of bias’ tool, use the Cochrane tool as the primary assessment of bias for interpreting results, choosing the primary analysis, and drawing conclusions. | Applicable to extracting and reporting risk of bias assessments for individual studies that were included in the included SRs. |
| Summarizing the findings | | | |
| 76 | Assessing the quality of the body of evidence | Use the five GRADE considerations (study limitations, consistency of effect, imprecision, indirectness and publication bias) to assess the quality of the body of evidence for each outcome, and to draw conclusions about the quality of evidence within the text of the review. | Extract quality of evidence assessments from the included SRs. Decide a priori what to do if quality of evidence assessments have not been performed or performed inconsistently across SRs. |
| 77 | Justifying assessments of the quality of the body of evidence | Justify and document all assessments of the quality of the body of evidence (for example downgrading or upgrading if using the GRADE tool). | Extract relevant information from the SRs. |
| Reaching conclusions | | | |
| 78 | Formulating implications for practice | Base conclusions only on findings from the synthesis (quantitative or narrative) of studies included in the review. | Directly applicable |
| 79 | Avoiding recommendations | Avoid providing recommendations for practice. | Directly applicable |

* The items listed are among those considered mandatory for Cochrane Intervention Reviews. The item numbers, names, and standards are from: Chandler J, Churchill R, Higgins J, Lasserson T, Tovey D. Methodological standards for the conduct of new Cochrane Intervention Reviews. Version 2.1, 8 December 2011. Available at http://www.editorial-unit.cochrane.org/sites/editorial-unit.cochrane.org/files/uploads/MECIR_conduct_standards%202.1.pdf

† The section from the above citation on “synthesizing the results of included studies” has been omitted from this table as it relates to the quantitative synthesis of individual studies in a meta-analysis. For the most part, overviews of reviews have been descriptive in nature. Guidance on performing indirect analyses or mixed treatment comparisons is beyond the scope of this paper.

Table 2. Preferred Reporting Items for Systematic Reviews and Meta-analyses (PRISMA) and their applicability to overviews of reviews*

| **Section/topic** | **Item No.** | **Checklist item** | **Applicability to overviews of reviews** |
| --- | --- | --- | --- |
| TITLE |  |  |  |
| Title | 1 | Identify the report as a systematic review, meta-analysis, or both. | Applicable. Identify the report as an “overview of reviews”. |
| ABSTRACT |  |  |  |
| Structured summary | 2 | Provide a structured summary including, as applicable: background; objectives; data sources; study eligibility criteria, participants, and interventions; study appraisal and synthesis methods; results; limitations; conclusions and implications of key findings; systematic review registration number. | Directly applicable |
| INTRODUCTION |  |  |  |
| Rationale | 3 | Describe the rationale for the review in the context of what is already known. | Directly applicable |
| Objectives | 4 | Provide an explicit statement of questions being addressed with reference to participants, interventions, comparisons, outcomes, and study design (PICOS). | Directly applicable |
| METHODS |  |  |  |
| Protocol and registration | 5 | Indicate if a review protocol exists, if and where it can be accessed (e.g., Web address), and, if available, provide registration information including registration number. | Directly applicable |
| Eligibility criteria | 6 | Specify study characteristics (e.g., PICOS, length of follow-up) and report characteristics (e.g., years considered, language, publication status) used as criteria for eligibility, giving rationale. | Directly applicable |
| Information sources | 7 | Describe all information sources (e.g., databases with dates of coverage, contact with study authors to identify additional studies) in the search and date last searched. | Directly applicable |
| Search | 8 | Present full electronic search strategy for at least one database, including any limits used, such that it could be repeated. | Directly applicable |
| Study selection | 9 | State the process for selecting studies (i.e., screening, eligibility, included in systematic review, and, if applicable, included in the meta-analysis). | Directly applicable |
| Data collection process | 10 | Describe method of data extraction from reports (e.g., piloted forms, independently, in duplicate) and any processes for obtaining and confirming data from investigators. | Directly applicable |
| Data items | 11 | List and define all variables for which data were sought (e.g., PICOS, funding sources) and any assumptions and simplifications made. | Directly applicable |
| Risk of bias in individual studies | 12 | Describe methods used for assessing risk of bias of individual studies (including specification of whether this was done at the study or outcome level), and how this information is to be used in any data synthesis. | Applicable. Overview authors should describe how they determined risk of bias for individual studies (e.g., from the SRs or from the individual studies), as well as how they assessed methodological quality of the included SRs. |
| Summary measures | 13 | State the principal summary measures (e.g., risk ratio, difference in means). | Applicable. Overview authors should state what summary measures were used in the included SRs and whether data were re-analyzed by overview authors to present consistent summary measures across SRs. |
| Synthesis of results | 14 | Describe the methods of handling data and combining results of studies, if done, including measures of consistency (e.g., I^2^) for each meta-analysis. | Partially applicable. Overview authors should describe their approach to synthesizing results from SRs. |
| Risk of bias across studies | 15 | Specify any assessment of risk of bias that may affect the cumulative evidence (e.g., publication bias, selective reporting within studies). | Directly applicable |
| Additional analyses | 16 | Describe methods of additional analyses (e.g., sensitivity or subgroup analyses, meta-regression), if done, indicating which were pre-specified. | Applicable. Overview authors should describe additional analyses done within the included SRs that were pre-specified to be of interest to the overview question. Overview authors should describe methods of additional analyses they performed. |
| RESULTS |  |  |  |
| Study selection | 17 | Give numbers of studies screened, assessed for eligibility, and included in the review, with reasons for exclusions at each stage, ideally with a flow diagram. | Directly applicable |
| Study characteristics | 18 | For each study, present characteristics for which data were extracted (e.g., study size, PICOS, follow-up period) and provide the citations. | Directly applicable |
| Risk of bias within studies | 19 | Present data on risk of bias of each study and, if available, any outcome level assessment (see item 12). | Applicable. Overview authors may want to summarize the risk of bias of studies contained within the included SRs. Overview authors should present data on methodological quality of each SR. |
| Results of individual studies | 20 | For all outcomes considered (benefits or harms), present, for each study: (a) simple summary data for each intervention group (b) effect estimates and confidence intervals, ideally with a forest plot. | Applicable. Overview authors should present for each included SR summary data for each intervention group and summary effect estimates and confidence intervals. |
| Synthesis of results | 21 | Present results of each meta-analysis done, including confidence intervals and measures of consistency. | Applicable. Overview authors should present results of meta-analyses that are relevant to their pre-defined question. |
| Risk of bias across studies | 22 | Present results of any assessment of risk of bias across studies (see Item 15). | Applicable (see item 19). Overview authors may want to summarize the risk of bias of studies contained within the included SRs. |
| Additional analysis | 23 | Give results of additional analyses, if done (e.g., sensitivity or subgroup analyses, meta-regression [see Item 16]). | Directly applicable |
| DISCUSSION |  |  |  |
| Summary of evidence | 24 | Summarize the main findings including the strength of evidence for each main outcome; consider their relevance to key groups (e.g., healthcare providers, users, and policy makers). | Directly applicable |
| Limitations | 25 | Discuss limitations at study and outcome level (e.g., risk of bias), and at review-level (e.g., incomplete retrieval of identified research, reporting bias). | Applicable, with focus on limitations at review-level. |
| Conclusions | 26 | Provide a general interpretation of the results in the context of other evidence, and implications for future research. | Directly applicable |
| FUNDING |  |  |  |
| Funding | 27 | Describe sources of funding for the systematic review and other support (e.g., supply of data); role of funders for the systematic review. | Directly applicable |

* The first three columns of this table are taken directly from: Moher D, Liberati A, Tetzlaff J, Altman DG, The PRISMA Group (2009). Preferred Reporting Items for Systematic Reviews and Meta-Analyses: The PRISMA Statement. PLoS Med 6(6): e1000097. doi:10.1371/journal.pmed1000097. The checklist containing the first three columns is also available at [www.prisma-statement.org](http://www.prisma-statement.org) and [www.equator-network.org](http://www.equator-network.org).
